# Chilean validation of the operationalized psychodynamic diagnosis-structure questionnaire (OPD-SQ) for personality structure

**DOI:** 10.1186/s40359-021-00640-4

**Published:** 2021-09-13

**Authors:** Nicolás Lorenzini, G. de la Parra, P. Dagnino, E. Gomez-Barris, C. Crempien, J. C. Ehrenthal

**Affiliations:** 1 Psychoanalysis Unit, Research Department of Clinical, Educational and Health Psychology, University College London, London, UK; 2 Department of Psychiatry, Faculty of Medicine, Pontificia Universidad Católica de Chile, Diagonal Paraguay 362, 8330077 Santiago, Chile; 3 Millenium Institute for Research on Depression and Personality – MIDAP, Santiago, Chile; 4 Faculty of Psychology, Universidad Alberto Hurtado, Santiago, Chile; 5 University of Cologne, Cologne, Germany

**Keywords:** Personality structure, Psychodynamic, Self-report, Operationalized psychodynamic diagnosis

## Abstract

**Background:**

This is the validation of the Operationalized Psychodynamic Diagnosis—Structure Questionnaire (OPD-SQ).

**Methods:**

A clinical sample of 399 adults and a nonclinical general population sample of 50 healthy adults completed measures of depression, attachment, psychiatric symptomatology and distress. Internal consistency and concurrent validity were assessed. Test–retest and Reliable Change Index were also calculated, as was the ability of the OPD-SQ to distinguish between the clinical and general population groups.

**Results:**

High internal consistencies were found; significant differences between clinical and nonclinical samples, and significant associations with psychiatric symptomatology, depression and psychological distress.

**Conclusion:**

The Chilean OPD-SQ has good reliability, and discriminates between clinical and healthy samples.

## Background

Traditional categorical and descriptive diagnoses, based on symptomatology as a solitary approach to mental disorder are increasingly being questioned. Complementary, more comprehensive systems aim at factors beyond symptomatology and also consider the patients’ strengths, their experience of the disorder, and other core aspects of psychopathology, in order to achieve a person-centred diagnosis [1]. Personality structure and characteristics are particularly important for this novel way of approaching psychopathology, for they comprise basic capacities and impairments in normal and impaired functioning. Broadly speaking, personality is here understood both as a predisposing factor for various mental disorders (a vulnerability factor), as well as a factor influencing the presentation of such disorders: severity of symptoms, subjective experience of the disorder and treatment response. In this paper we present the OPD-SQ, a self-report instrument that allows for a dimensional evaluation of personality functioning.

### Personality functioning and psychopathology

International research on dimensional models of personality functioning was considerably increased since the formulation of Criterion A (Levels of Personality Functioning; LPFS) of the Alternative Model of Personality Disorders (AMPD) of the DSM-5, which comprises four domains in two broad areas of functioning regarding the self (identity, self-direction) as well as interpersonal functioning (empathy, intimacy) [2, 3]. Contemporary studies point towards the conclusion that LPFS can be reliably measured and it shows validity in a variety of measures of psychopathology [4]. Differently from the the World Health Organization’s International Statistical Classification of Diseases and Related Health Problems (11th ed.; ICD-11) [44], the fifth version of the Diagnostic and Statistical Manual of Mental Disorders (DSM 5) [5] did not evolve towards a formal endorsement of a dimensional approach of personality and its disorders, but rather moved those perspectives into Section III (“Emerging Measures and Models”). The ICD-11 from the World Health Organization (WHO) went one step further. Similar to the DSM-5 proposal, personality disorders will be diagnosed on a severity scale which includes basic problems or vulnerabilities in the areas of self and interpersonal functioning [6].

The models formulated by the DSM-5 AMPD and ICD-11 are based on a rich tradition of mostly psychodynamic and interpersonal approaches to measure personality functioning or integration. For example, this dimensional perspective is shared among several measurement instruments like the Shedler-Westen Assessment Procedure (SWAP) [7], the Structured Interview of Personality Organization (STIPO) [8], which follows Otto Kernberg’s model, or the Mental Functioning Dimension of the Psychodynamic Diagnostic Manual [9]. A similar perspective was proposed by the OPD-2 in 2006 [10]. Here personality functioning is also conceptualized as dimensional rather than categorical, with a description of a number of areas of personality functioning to be evaluated independently in order to facilitate therapeutic interventions (see Table 1). For example: a particular functional vulnerability can be discovered and prioritised in psychotherapy (e.g. vulnerability or deficit in impulse control), using the support of healthy functional strengths (e.g. self-perception). For a comparison between the LPFS and the OPD-2, including the relationship between the OPD and DSM and ICD, see Zimmermann et al. [11].

**Table 1:** Structural personality functions according to the Axis IV of the OPD-2

Domain	Function	Sub-function
Perception/cognition	Self-perception	Self-reflection
		Affect differentiation
		Identity
	Object perception	Self-object differentiation
		Whole object perception
		Realistic object perception
Regulation	Self-regulation	Impulse control
		Affect tolerance
		Regulation of self-esteem
	Regulation of relationships	Protecting relationships
		Balancing interests
		Anticipation
Communication	Internal communication	Experiencing affect
		Use of fantasies
		Bodily-self
	External communication	Making contact
		Communicating affect
		Empathy
Attachment	Attachment to internal objects	Internalization
		Utilizing introjects
		Variability of attachment
	Attachment to external objects	Capacity for attachment
		Accepting help
		Detaching from relationships

Most of these measures, including the OPD-2 in its original version, require a manual and a trained observer for their administration and evaluation. Despite their reliability, the administration of the tests (1–2 h interview) and the training of the raters are time consuming and costly [10]. A self-report instrument is a valuable contribution to the research of personality structure, but also to clinical practice, because it allows to find a common language with the patient that closely resembles their actual experience, values the patient's perspective and can be therefore used for joint treatment planning [12, 13].

### Operationalized psychodynamic diagnosis: rationale

The OPD-2 is the base of the present self-report questionnaire (OPD-SQ), therefore its rationale and functioning must be well understood before introducing the self-report itself. The OPD-2 brings together descriptive and symptomatologic criteria with clinically-relevant psychodynamic domains, which guide the clinician in the indication and planning of psychotherapy, and allow for the specific evaluation of changes achieved by psychotherapeutic interventions. The OPD-2 organizes diagnostic information in five axes: Axis I: Experience of Illness and Prerequisites for Treatment; Axis II: Interpersonal Relations; Axis III: Conflict; Axis IV: Structure and; Axis V: Psychic and Psychosomatic Disorders [10, 11]. In what follows, we limit our description to Axis IV, namely the Levels of Structural Integration. (LSIA; For detailed descriptions of Axes I-III and V, see [10, 14]).

Psychic structure evolves around two lifelong tasks, the development of capacities for interpersonal relatedness and the development of self-definition or identity, underpinned by functions oriented towards self-regulation and the relationship between the self and its internal and external objects. Impaired structure is usually rooted in adverse developmental conditions, i.e. experiences of abuse or neglect, which compromise the acquisition of these and other capacities. While personality structure itself is conceived by the developers of the OPD-2 as the overall organization or arrangement of mental dispositions, its manifestations can be observed and described in a variety of domains, functions and sub-functions, listed in Table 1:

The Perception/cognition domain includes the structural functions Self-perception and Object Perception. These refer to the ability to differentially perceive an image of the self and its intrapsychic events, especially affects, as well as the ability to develop a realistic image of the other.

The Regulation domain includes the functions of Self-regulation and Regulation of Relationships. The first is an integrative aspect of the psychic experience that results in the capacity to take responsibility for one's own actions. The tasks of Regulation of Relationships include both the protection of relationships against one's own impulses, as well as the safeguarding of one's own interests, so that these are not lost to the influence of others.

The Communication domain includes Internal Communication and External Communication The first refers to the ability to carry on internal dialogues and to understand oneself. The ability to let emotions emerge in oneself and to live them is a prerequisite to achieve this type of communication. External Communication points to emotional exchanges between the self and the other: the establishment of emotional contact between people, the ability to communicate one's own affects, the capacity to let oneself to be affected by others’ emotional experiences, as well as the mutual understanding and the feeling of the "we" of reciprocity.

The Attachment domain includes the functions Attachment to Internal Objects and Attachment to External Objects. Both point to the abilities to relate to others, both intrapsychically and in interpersonal contact. The first is the capacity to create internal representations of significant others, which in times of distress could provide support and comfort. It also refers to the capacity to become emotionally attached to others in real relationships, to separate and to mourn.

A well-integrated personality structure fosters the creative and flexible availability of regulatory and adaptive psychic functions, allowing for a homeostatic equilibrium which is dynamic, not rigid or immutable. It is at the same time the basis for dealing with developmental tasks across the life-span, integrating new information to establish new regulatory rules and to modify existing ones [10]. The LSIA is usually rated by trained experts and has received considerable empirical support regarding its reliability and validity [11]. In addition, the OPD system is widely used in German-speaking countries' health-care systems, with more than 25 years of experience in training and clinical application.

In summary, the evaluation of this Axis allows not only for the assessment of the structural integration of personality in a continuum from functional to dysfunctional, but also to appraise the specific vulnerabilities and strengths of an individual, thus facilitating the planning of psychotherapeutic interventions and the identification of deficits implicated in various psychopathologies within a research context.

#### The operationalized psychodynamic diagnosis: structure questionnaire (OPD-SQ)

This article introduces the Spanish-language version of the Operationalized Psychodynamic Diagnosis—Structure Questionnaire (OPD-SQ) [12], originally developed and published in German. It is a self-report questionnaire that measures several different dimensions related to structural abilities and vulnerabilities, following the rationale of the Axis IV of the Operationalized Psychodynamic Diagnosis System (OPD-2) [10]. The OPD-SQ is based on the definition of OPD Levels of Structural Integration described above. Through its 95 items, where participants indicate the degree to which they identify with each statement, the questionnaire evaluates personality function by measuring four personality domains, each oriented towards the self or towards others. These domains were described above: (a) Perception/cognition, (b) Regulation, (c) Emotional communication, and (d) Attachment. The original German version of this measure reported internal consistencies between 0.72 and 0.91 for its various scales in several samples [12].

This self-report consists of 95 items divided in four scales. Each dimension is represented in their polarities self-other, giving the OPD-SQ a total of 8 subscales. Each of these 95 items is a statement followed by a Likert scale, where the participant must indicate their level of agreement with the statement (0 = totally disagree; 4 = totally agree). The items and scales are organised as following:

1. Cognitive abilities: composed of 29 statements divided into the subscale Self-Perception (12 items) and the subscale Object Perception (17 items).
2. Regulation abilities: twenty-five affirmations divided into Self-Regulation (13 items) and Regulation of Relationships (12 items).
3. Communication abilities: Made of 25 statements divided into Emotional Internal Communication (11 items) and Emotional External Communication (14 items).
4. Attachment abilities: Comprising 16 statements within the subscales of Attachment to Internal Objects (8 items) and Attachment to External Objects (8 items).

The authors of the original German measure allowed for maximum one missing response on each scale to rate the questionnaire during validation (in clinical practice, more missing responses could be allowed, depending on the content of those unanswered questions). The questionnaire yields partial scores for each scale and subscale, and a total score for the structural functioning of the subject. Higher scores represent worse functioning. Table 2 details the sub-scales of the OPD-SQ. Although the original version of the questionnaire was constructed from a theoretical perspective, and therefore did not test factorial structures of the instrument, subsequent research provides information about different factorial models. For example, Ehrenthal et al. [20] found three highly correlated factors in exploratory factor analyses for the development of a German short version, which showed a good model fit in confirmatory analyses as well. Zimmermann et al. [17] found a similar underlying structure. Later studies specifically tested different factorial structures. Obbarius et al., (in press) found EFA-models with bifactor rotations including seven-, eight-, and nine-factor-solutions to have acceptable to good model fit indexes, but also confirmed a strong general factor. In three studies from Jauk and Ehrenthal [43], models of a general latent factor resulting from the four large areas of perception, regulation, communication, and the ability to attach showed satisfactory to good model fit indexes. Summarizing the findings, depending on the assumed latent structures being tested, several models seem to converge well, indicating the need for a strong theoretical basis for a specific test.

**Table 2:** Summary of the OPD-SQ subscales, sub-functions assessed, example items (translated from Spanish for illustration) and internal consistency (Cronbach's <)

Subscale	Sub-function included	No. of items	Example item	Internal consistency (Cronbach’s α)		
				Gral population sample	Clinical sample	Total sample
Self-Perception	Reflection of self	4	I find very difficult to describe myself	.865	.915	.923
	Differentiation of affects	4	I often don’t know very well how I am feeling			
	Identity	4	Sometimes I feel or do things that do not match with myself			
Object Perception	Self-object differentiation	7	Sometimes I doubt whether someone else is thinking something about me, or if it just my imagination	.792	.865	.874
	Holistic object perception	4	If the other person is not in my same mood, we will not work-out			
	Realistic object perception	6	People tell me that I always end up picking the wrong friends			
Self-Regulation	Regulation of impulse	4	Sometimes I get so angry that I do not respond for my actions	.725	.874	.883
	Tolerance of affects	5	Sometimes my emotions are so strong that they scare me			
	Self-Regulation-esteem	4	I find it difficult to overcome when someone criticizes me			
Regulation of Relationships	Regulation of Relationships	6	When I am angry I tend to damage my relationships	.727	.850	.851
	Anticipation	6	Sometimes I misjudge how my behavior affects others			
Internal Communication	Experiencing of affects	4	It is difficult to perceive my own emotions	.550	.767	.780
	Utilizing fantasies	3	My fantasies and ideas vitalize and enrich me			
	Body-self	4	I am often incapable of perceiving well my body			
External Communication	Establishing contact	4	I find it difficult to establish contact with other people	.672	.685	.684
	Communicating affects	6	I have been told that I do not show my feelings			
	Empathy	4	When someone is having a bad time, I tend to worry			
Attachment to Internal Objects	Internalization	4	I often think of certain people who could harm me	.703	.835	.842
	Utilizing introjects	4	I find it difficult to do something good for myself			
Attachment to External Objects	Accepting help	4	I find it difficult to ask others for help	.477	.677	.682
	Dissolving attachment	4	Separations and goodbyes are difficult to me			

The original German questionnaire [12] found high positive correlations with general psychopathology, attachment insecurity as well as neuroticism, and negative with Openness, Agreeableness and Conscientiousness. The instrument was able to discriminate between a general population sample and a clinical sample comprising both ambulatory and hospitalized patients with a high effect size (Cohen’s d = 1.50). It yielded good internal consistencies in all scales (Cronbach ‹ between 0.72 and 0.91). Subsequent research found a significant positive correlation (r = 0.62) between OPD-SQ and OPD-LSIA expert-ratings and incremental validity in predicting the number, comorbidity and severity of DSM personality disorders [11, 13]. It differentiates between depressed patients with vs. without a comorbid diagnosis of borderline personality disorder [15], and it is associated -over and above a categorical diagnosis-with negative affectivity in inpatients in psychotherapy [16]. The OPD-SQ is significantly associated with other measures of personality dysfunction, including the General Assessment of Personality Disorder (GAPD) [17], and other questionnaires [18], including trait- and performance-based measures of emotional experience and a high association with the Level of Personality Functioning Scale—Self Report [19]. A 12 item screening version of the OPD-SQ [20] was also related (r = 0.78) to LPFS expert-ratings and to reflective functioning [21]. From a clinical perspective, OPD-SQ scores showed relevant associations with slopes of plasma glucose in type 2 diabetes patients [22], eating disorder profiles [23], bipolar disorder [24], trauma symptom severity [25], to name some areas of research. A preliminary study on the present Chilean version [26] reported good to excellent internal consistencies in all scales (Cronbach ‹ between 0.71 and 0.93), and was able to discriminate between healthy and patient samples (d = 1.05). It also showed positive correlations with psychological distress measured with the Outcome Questionnaire—45 Item version (OQ-45) [27] and with depressive symptomatology measured with the Beck Depression Inventory (BDI-I) [28].

The present study aims at delivering a more complete validation of the OPD-SQ. It is hypothesized that this measure will not only show satisfactory psychometric indexes for reliability and validity, but also that it will discriminate between our clinical and nonclinical samples.

## Methods

### Participants

Our sample consisted of 449 adult participants of which 50 were healthy participants from the general population (11.14%). The clinical sample was composed of 399 patients attending mental health services at 22 different primary health centres in Santiago’s Metropolitan Area. The sample was collected thanks to different research projects whose common factor was research on depression. Therefore, the inclusion criteria for the clinical sample was scoring above the Chilean cut-off for depression in the Beck Depression Inventory (a score of 14 or above). On the other hand, the exclusion criteria were: age under 18, those seeking treatment for substance use issues, and those with psychotic symptoms, cognitive dysfunction or eating disorder (as in previous studies) [46]. All patients were informed of the study and invited to participate during their first clinical appointment. The general population sample were adults who also lived in Santiago. They were contacted through 27 volunteers of this study (each volunteer could invite a maximum of three participants), and invited to participate in a study related to depression and personality. Exclusion criteria were current psychological or psychiatric treatment or the intention of starting treatment. All participants provided signed consent. Table 3 shows the demographic characteristics of our sample. Samples were comparable in gender and age, but the clinical sample had a slightly larger proportion of participants with university degrees.

**Table 3:** Demographic characteristics of this study's sample

	General population sample	Clinical sample	Total sample
Age: mean (SD)	34.42 (14.49)	36.20 (13.72)	35.97 (13.81)
Gender: n(% of female)	30 (60.00%)	287 (76.33%)	317 (74.41%)
Education n (%)			
Illiterate	–	–	–
Primary school uncompleted	–	4 (1.25%)	4 (1.03%)
Primary school completed	2 (4.00%)	9 (2.82%)	11 (2.84%)
High school uncompleted	2 (4.00%)	17 (5.33%)	19 (4.90%)
High school completed	15 (30.00%)	96 (30.09%)	111 (30.08%)
Technical college completed	14 (28.00%)	65 (20.38%)	79 (21.41%)
University or higher degree	17 (43.00%)	128 (40.12%)	145 (39.30%)
Occupation n (%)			
Housewife	9 (18.00%)	37 (12.21%)	46 (13.03%)
Student	20 (40.00%)	87 (28.71%)	107 (30.31%)
Employee	14 (28.00%)	97 (32.01%)	111 (31.44%)
Freelancer	6 (12.00%)	27 (8.91%)	33 (9.35%)
Unemployed	–	19 (6.27%)	19 (5.38%)
Not working for medical reason	–	23 (7.59%)	15 (4.25%)
Retired	1 (2.00%)	9 (2.97%)	18 (5.10%)
Other	–	4 (1.32%)	4 (1.13%)

### Instruments

#### Beck depression inventory (BDI-I)

Self-report measuring depressive symptomatology through 21 items [28, 29]. It has been translated and validated in several countries, including Chile [30]. Each item is scored from 0 to 3 (e.g. Item 1, score 0 = “I do not feel sad”; score 3 = “I am so sad and unhappy that I can’t stand it”). The version used has excellent internal consistency (Cronbach’s α = 0.92). The Chilean cut-off point for the diagnosis of depression is a score of 14 or above [31].

#### Experiences in close relationships scale (ECR)

It assesses the attachment style of individuals in their romantic relationships [32]. Two dimensions comprise the scale: (i) anxiety and (ii) avoidance. This scale has been used to measure attachment in Chilean samples, reaching reliability indexes of 0.84 for the anxiety scale and 0.83 for the avoidance scale [33].

#### Operationalized psychodynamic diagnosis: structure questionnaire (OPD-SQ)

A 95-item self-report to measure personality function, described in the introduction to this paper. An English language version can be found here: http://strukturdiagnostik.de/international-versions/. The translation and back-translation into Spanish was carried out in close collaboration between the Chilean team (partly authors of the present work) and the creators of the instrument in Germany [26]. This process, which involved bilingual professionals in Chile and Germany, included the following stages: First, translation of the instrument by a bilingual psychologist and psychiatrist who was in charge of the translation of the OPD-2 manual into Spanish; intelligibility review by a non-psychologist with knowledge of German, technical discussion of each item by the authors of the present paper, delivery of the instruments to five bilingual Chilean judges to compare the Spanish version with the German version; and delivery of five versions with corrections which were unified by three Chilean OPD experts. As a result of this process up to this point, this second Spanish version was sent to the creators of the instrument in Germany for reverse translation, which was compared with the original version. Subsequently, the Chilean and German teams entered into a discussion and analysis process, where it was necessary to unify criteria regarding the level of intensity of the adjectives, or terms that alluded to cognitive or affective processes. In this discussion between the teams it was necessary to rely on an English version of the instrument. This process finally resulted in a third version that was given for comprehensibility analysis to twelve psychologists and psychiatrists in Chile, as well as to some people not related to the subject. After a final adjustment resulting from this review, a pilot application of the instrument is made to a group of Chilean consultants of medium–low socioeconomic levels, having to make some adjustments that result in a fourth version, which is sent to Germany for a final review and discussion by both groups, resulting in the final version.

#### Outcome questionnaire (OQ-45.2)

A 45-item self-report developed by Lambert et al. [27]. It measures current psychological distress in three areas of functioning, namely symptomatic (25 items; e.g.: “I tire quickly”), interpersonal relations (11 items; e.g.: “I feel unhappy in my marriage/significant relationship”) and social role (5 items; e.g.: I am not working/studying as well as I used to”). The Chilean version used has excellent internal consistency (Cronbach’s α = 0.93), good test–retest reliability (r = 0.84) and positive correlations with similar instruments (0.53–0.88). It is sensitive to psychopathology and change [27, 34, 35]. The Chilean version considers a score of 73 or more as clinical cut-off, and a change score of 17 as Reliable Change Index (RCI) [36].

#### Symptom checklist, revised (SCL-90-R)

A 90-item self-report designed to evaluate a broad range of psychological problems and symptoms of psychopathology [37]. It takes 12–15 min to administer, yielding nine scores along primary symptom dimensions and three scores of global distress. Symptoms assessed are somatization, obsessive–compulsive, interpersonal sensitivity, depression, anxiety, hostility, phobic anxiety, paranoid ideation and psychoticism. The three global indexes are Global Severity Index (GSI), Positive Symptom Distress (PSDI), and Positive Symptom Total (PST). The Chilean version used in this study has reported good internal consistency (α = 0.64 to 0.82 for the various scales), and there are thorough norm values for the country’s population [38].

### Procedure

This study counts with ethical approvals from the Bioethics Committee of the Southeast Metropolitan Health Service in Santiago and the Ethics Committees of the Psychology School of the Pontificia Universidad Católica de Chile, Universidad de la Frontera, Universidad del Desarrollo, Universidad Gabriela Mistral and Universidad Alberto Hurtado. Participation was voluntary for the clinical sample. Patients were informed that their treatment would not be compromised by their decision to participate. Participants from the general population received a compensation of approx. USD $15 for two research sessions: the first one consisted in the collection of demographic data, OPD-SQ, BDI and OQ-45. One month later, participants returned to complete the OPD-SQ again. All participants signed an informed consent letter. Once this letter was signed, participants completed the self-report measures in presence of a research assistant, in case they had doubts about the instruments.

### Analysis plan

Data were analyzed using SPSS 25 [39]. We have followed the analysis plan for the initial validation of the original German measure [12]. For internal consistency, Cronbach’s alphas were calculated for the total measure and subscales, in the clinical, general population and total samples. Nonparametric Spearman correlation coefficients were calculated between the scales of the OPD-SQ. Means and standard deviations were calculated for each scale and sample. One-month test–retest reliability was calculated using Spearman correlations in the nonclinical sample, given that they did not receive psychotherapy during that month, and that the nonclinical sample size is adequate to achieve a > 0.8 power for these two measurement points [45]. The Reliable Change index was then calculated, together with the clinical cut-off point for each scale, according to the method suggested by Jacobson and Truax [40], namely by calculating the difference between participants pre-test and post-test scores, divided by the standard error of that difference. To ascertain whether the OPD-SQ distinguished between clinical and healthy samples, Mann–Whitney U tests were used, for scores were not normally distributed according to previous Kolmogorov–Smirnov tests. Concurrent validity was assessed using Spearman bivariate correlations between the OPD-SQ and other measures.

## Results

### Internal consistency

The 95 items of the OPD-SQ showed an excellent internal consistency (α = 0.97) for the full sample. The clinical sample showed an α = 0.97 while the general population sample yielded an α = 0.93, both excellent.

Internal consistencies of the OPD-SQ scales are listed in Table 2. They ranged between α = 0.92 and α = 0.68 for the full sample, between α = 0.91 and α = 0.67 for the clinical sample and between α = 0.87 and α = 0.48 for the general population sample. Table 4 shows that all scales of the OPD-SQ were positively and significantly correlated to each other.

**Table 4:** Spearman correlation coefficients between the scales of the OPD-SQ

	2	3	4	5	6	7	8
1. Self-perception	.793	.863	.712	.782	.438	.817	.610
2. Object perception		.757	.759	.653	.445	.779	.621
3. Self-regulation			.780	.713	.435	.801	.640
4. Regulation of relationships				.587	.520	.726	.534
5. Internal communication					.402	.728	.513
6. External communication						.440	.306
7. Attachment to internal objects							.646
8. Attachment to external objects							

All coefficients are significant at p < .001

### Test–retest reliability

The nonclinical sample (n = 50) was retested after one month (mean days = 31.55; SD = 3.86). The total score for the OPD-SQ showed good test–retest reliability (rho = 0.87; p < 0.001). All subscales showed acceptable to good test–retest indexes ranging between rho = 7.22 and rho = 0.82 (all p < 0.001). The Reliable Change Index for this sample was 0.44.

### Discrimination between clinical and nonclinical samples

Table 5 shows the means and standard deviations of all measures used in the study grouped by subsample. Regarding depressive symptomatology, the clinical sample is above the score of 14 proposed as clinical cut-off for Chilean adults [28, 31], and it is significantly higher than the general population sample (U = 2294.50; p < 0.001; d = 0.93).

**Table 5:** Descriptive statistics and clinical differences of this study's measures

Measure	Gral population		Clinical		U	D	Clinical cut-off
	Mean	SD	Mean	SD			
BDI	7.20	5.80	22.76	11.76	2294.50**	0.93	
OPD-SQ							
Total score	1.35	0.44	2.14	0.72	2885.00**	0.81	1.65
Self-perception	1.14	0.73	2.42	1.11	2782.50**	0.83	1.65
Object perception	1.19	0.58	2.00	0.85	3355.00**	0.71	1.52
Self-regulation	1.26	0.57	2.32	0.94	2756.00**	0.83	1.66
Regulation of relationships	1.35	0.61	2.05	0.88	4020.00**	0.59	1.64
Internal communication	1.15	0.47	1.91	0.79	3401.00**	0.70	1.43
External communication	1.41	0.50	1.58	0.62	6367.00	0.20	1.49
Attachment to internal objects	1.34	0.69	2.25	1.01	3454.00**	0.70	1.71
Attachment to external objects	1.99	0.57	2.61	0.76	3942.00**	0.63	2.26
OQ-45							
Total score	47.74	17.74	86.72	28.74	2503.00**	0.90	
Symptom distress	27.78	11.05	53.49	19.16	2464.00**	0.90	
Interpersonal relations	10.66	4.82	18.37	7.08	3470.00**	0.75	
Social role	9.30	3.89	14.87	5.66	3940.00**	0.68	
ECR^c^							
Anxiety			3.87	1.25			
Avoidance			4.16	0.75			
SCL-90-R^c^							
Somatization			1.66	0.89			
Obsessive–compulsive			2.08	0.92			
Interpersonal sensitivity			1.55	0.96			
Depression			2.00	0.99			
Anxiety			1.69	0.96			
Hostility			1.00	0.79			
Phobic anxiety			1.10	0.97			
Paranoid ideation			1.27	0.89			
Psychoticism			1.30	0.78			
Global severity index (GSI)			1.60	0.78			
Positive symptom total (PST)			57.35	20.47			
Positive symptom distress (PSDI)			2.34	0.56			

BDI: Beck Depression Inventory; OPD-SQ: Operationalized Psychodynamic Diagnosis – Structure Questionnaire; OQ-45: Outcome Questionnaire 45; ECR: Experience in Close Relationships; U: Mann–Whitney U test, d: Cohen’s d; *p < .05; **p < .001; ^c^only clinical participants; ^n^clinical participants (n = 15);^m^clinical participants (n = 14)

Likewise, the mean of the clinical sample is above the Chilean cut-off for the OQ-45 (73 or above), and significantly different from the nonclinical sample (U = 2503.50; p < 0.001; d = 0.90).

The OPD-SQ was also able to distinguish between clinical and nonclinical samples, using either the total score or each one of the scales. This is consistent with the authors’ hypotheses: a clinical sample is expected to show a lower level of structural functioning, as it is shown in Table 5. The table also lists the calculated clinical cut-off scores for this sample in each scale of the OPD-SQ. The clinical cut-off for the total score of the OPD-SQ was 1.65.

### Relationship between the OPD-SQ and other measures

Correlations of the OPD-SQ and measures of attachment, psychological distress (OQ-45), depressive symptomatology (BDI) and general psychopathology (SCL-90-R) are shown in Table 6. These significant and positive correlations suggest that increased structural functioning impairment is associated with more severe general and depressive symptomatology, and with psychological distress. In the case of attachment anxiety, correlations with all scales of the OPD-SQ are positive and significant. While with attachment avoidance relationships are generally inverse, with the exception of Object Perception.

**Table 6:** Spearman correlations between the OPD-SQ and measures of psychological distress, depression symptoms and attachment

	OPD-SQ total	Self-perception	Object perception	Self-regulation	Regulation of relationships	Internal communication	External communication	Attachment to internal objects	Attachment to external objects
OQ-45 total	0.767**	0.738**	0.608**	0.742**	0.615**	0.686**	0.328**	0.718**	0.547**
OQ SD	0.780**	0.769**	0.624**	0.761**	0.595**	0.688**	0.314**	0.728**	0.578**
OQ IR	0.633**	0.577**	0.502**	0.586**	0.569**	0.587**	0.317**	0.587**	0.426**
OQ SR	0.527**	0.494**	0.405**	0.517**	0.431**	0.471**	0.241**	0.507**	0.363**
BDI	0.649**	0.657**	0.547**	0.631**	0.480**	0.569**	0.226**	0.625**	0.505**
ECR Anxiety^c^	0.429**	0.438**	0.420**	0.460**	0.353**	0.207*	0.433**	0.301**	0.468**
ECR Avoidance^c^	-0.239**	-0.189*	0.292**	-0.185**	-0.193*	-0.089	-0.165	-0.130	-0.228**
SCL Somatization^c^	0.478**	0.454**	0.434**	0.408**	0.364**	0.489**	0.279**	0.469**	0.353**
SCL Obsessive-compulsive^c^	0.580**	0.623**	0.474**	0.630**	0.600**	0.631**	0.561**	0.639**	0.452**
SCL Interpersonal Sensitivity^c^	0.697**	0.653**	0.559**	0.527**	0.461**	0.576**	0.236*	0.572**	0.381**
SCL Depression^c^	0.641**	0.624**	0.507**	0.578**	0.520**	0.614**	0.361**	0.637**	0.459**
SCL Anxiety^c^	0.595**	0.578**	0.468**	0.587**	0.483**	0.557**	0.358**	0.583**	0.389**
SCL Hostility^c^	0.427**	0.386**	0.378**	0.442**	0.377**	0.363**	0.285**	0.340**	0.333**
SCL Phobic Anxiety^c^	0.589**	0.611**	0.411**	0.548**	0.443**	0.599**	0.416**	0.532**	0.404**
SCL Paranoid Ideation^c^	0.578**	0.563**	0.536**	0.501**	0.461**	0.544**	0.375**	0.535**	0.368**
SCL Psychoticism^c^	0.715**	0.689**	0.605**	0.649**	0.592**	0.629**	0.458**	0.664**	0.499**
SCL GSI^c^	0.688**	0.675**	0.556**	0.639**	0.553**	0.646**	0.417**	0.658**	0.475**
SCL PST^c^	0.663**	0.647**	0.565**	0.590**	0.544**	0.653**	0.460**	0.626**	0.413**
SCL PSDI^c^	0.544**	0.528**	0.414**	0.548**	0.427**	0.466**	0.244**	0.528**	0.458**

SD: Symptom Distress; IR: Interpersonal Relations; SR: Social Role. BDI: Beck Depression Inventory; ECR: Experiences in Close Relationships; SCL: Symptom Checklist; GSI: Global Severity Index; PST: Positive Symptom Total; PDSI: Positive Symptom Distress; *p < 0.05; **p < 0.01; ^c^only clinical sample

## Discussion

This study has shown the psychometric properties of the OPD-SQ [12] in its Spanish-language version for use in Chilean populations. The instrument showed good levels of internal consistency, it was able to distinguish clinical from nonclinical samples and yielded significant positive correlations with measures of depressive symptomatology and psychological distress. The same pattern of results was found for each one of the 8 scales comprising the OPD-SQ. All these results are in line with the theoretical tenets of this questionnaire: an impoverished personality function is expected to be associated with increased severity of symptoms and psychological distress. Likewise, a higher level of personality functioning is theoretically, a marker for mental health.

The OPD-SQ is akin to other similar instruments. An important one among those is the Level of Personality Functioning Scale (LPFS) del AMPD del DSM-5 [5]. Both are based on a psychodynamic perspective that understands the development of personality structure as a process focused on the maturation of the self, on the one hand, and the capacity to relate to others, on the other. Such development can be hindered; both instruments consider these hindrances as reflected in personality dysfunctions that can be quantified. Accordingly, the LPFS proposes four domains to be assessed: identity, self-direction, empathy and intimacy. The functioning of these domains is differentiated in 5 levels from no impairment to extreme impairment. Therefore, the comparison between these both models of assessment of personality structure functioning is relevant [11]. This comparison was made by Zimmermann et al. [11] using a judge-based assessment. They found that the LPFS of the AMPD was less sensitive to differentiate low-integrated vs. disintegrated structures, and therefore less sensitive to detect more severe pathology. These authors also found a significant overlap between the OPD subdimensions and the LPFS domains; at the same time there are features that were not covered or rarely covered by the LPFS with respect to the OPD: the use of fantasy, the experience of the bodily self, the ability to communicate affect, internalization, use of introjects, variability of attachment, accepting help and detaching from relationships (see subfunctions in Table 1), i.e. according to the results of these authors, the LPFS captures less the functions related to communication and attachment when compared to the OPD, which would have consequences in its ability to predict personality disorder. However, if we look comparatively at both scales, we see that the OPD system falls short with respect to those functions that have to do with agency and that are included in the self-direction domain of the LPFS. Thus, the OPD-SQ may not capture the vulnerability of those patients who have difficulties in making a plan, meeting goals and/or prosocial standards.

Mental health is influenced by individual, contextual variables and their interaction [41]. Notwithstanding the focus of this study being individual vulnerability (specifically personality structure), it is necessary to keep in mind the role of social and contextual sources of vulnerability (like poverty, chronic physical health issues, exposure to violence, discrimination and inequality) in the development and course of psychological problems, like depression. Future research should look into the interaction between those social determinants and personality structural function.

Limitations of the present study include the lack of factorial analysis, concerning the possible factor structure of the Chilean version of the OPD-SQ, but we decided to follow the original German validation paper and assess reliability and validity with more basic indexes [12]. A thorough test of confirmatory models of assumed factor structures is an important project for future research. However, this would need a larger sample size, a comparison sample (e.g. from Germany) and assumptions on variables that may explain differences (e.g. from a perspective of transcultural psychiatry).

A further limitation relates to the clinical use of the OPD-SQ. While this instrument can be used both in research and clinical context, further research is needed to ascertain the correspondence between information yielded by the OPD-SQ and that obtained by coding the OPD-2 interview in a larger sample, in order to evaluate the potential of this self-report to guide therapeutic decisions. This would allow not only to elucidate the association between OPD-based instruments, but also to explore the similarities between self-observation and the observations made by a trained professional.

## Conclusions

With this study, we are delivering to the Spanish-speaking community an instrument that measures the functioning of personality structure in eight different dimensions, according to the OPD system, in a reliable and valid manner according to scientific parameters. At a time of an important development of Ibero-American psychotherapy research, this instrument fills a gap for researchers interested in the study of personality disorders, especially if a dimensional and psychodynamic approach to the subject is used. Remaining interesting challenges are the study of the different functions’ potential for psychotherapeutic change, which of them are more sensitive to which psychotherapeutic interventions and at what point in the process. Another line of development will be to continue developing studies of the structural functioning underlying depression and the introjective (self-critical) and anaclitic (dependent) premorbid depressive styles, as the authors of this paper have been doing [47, 48].

We agree with Zimmermann et al. [11] when they talk about the “relatively broad consensus that a dimensional rating of the severity of personality dysfunction is central for the future assessment of Personality Disorder” (p. 1). In this sense,this instrument is in line with the contemporary direction towards more functional and dimensional diagnosis in mental health, for the benefit of our individual patients [42].

## Data Availability

The datasets used and/or analysed during the current study are available from the corresponding author on reasonable request.
